# Defining Key Performance Indicators for the California COVID-19 Exposure Notification System (CA Notify)

**DOI:** 10.1177/00333549221129354

**Published:** 2022-11

**Authors:** Eliah Aronoff-Spencer, Camille Nebeker, Alexander T. Wenzel, Kevin Nguyen, Rachel Kunowski, Mingjia Zhu, Gary Adamos, Ravi Goyal, Sepideh Mazrouee, Aaron Reyes, Nicole May, Holly Howard, Christopher A. Longhurst, Mohsen Malekinejad

**Affiliations:** 1 Division of Infectious Diseases and Global Public Health, School of Medicine, University of California San Diego, La Jolla, CA, USA; 2 University of California San Diego Health, La Jolla, CA, USA; 3 The Design Lab, University of California San Diego, La Jolla, CA, USA; 4 Herbert Wertheim School of Public Health and Human Longevity Science, University of California San Diego, La Jolla, CA, USA; 5 Department of Biomedical Informatics, School of Medicine, University of California San Diego, La Jolla, CA, USA; 6 California Connected, Center for Infectious Diseases, California Department of Public Health, Richmond, CA, USA; 7 Institute for Global Health Sciences, University of California San Francisco, San Francisco, CA, USA; 8 Department of Pediatrics, School of Medicine, University of California San Diego, La Jolla, CA, USA; 9 Department of Epidemiology and Biostatistics, University of California San Francisco, San Francisco, CA, USA

**Keywords:** digital exposure notification, universal design, contact tracing, internet-based intervention, digital technology, COVID-19

## Abstract

**Objectives:**

Toward common methods for system monitoring and evaluation, we proposed a key performance indicator framework and discussed lessons learned while implementing a statewide exposure notification (EN) system in California during the COVID-19 epidemic.

**Materials and Methods:**

California deployed the Google Apple Exposure Notification framework, branded CA Notify, on December 10, 2020, to supplement traditional COVID-19 contact tracing programs. For system evaluation, we defined 6 key performance indicators: adoption, retention, sharing of unique codes, identification of potential contacts, behavior change, and impact. We aggregated and analyzed data from December 10, 2020, to July 1, 2021, in compliance with the CA Notify privacy policy.

**Results:**

We estimated CA Notify adoption at nearly 11 million smartphone activations during the study period. Among 1 654 201 CA Notify users who received a positive test result for SARS-CoV-2, 446 634 (27%) shared their unique code, leading to ENs for other CA Notify users who were in close proximity to the SARS-CoV-2–positive individual. We identified at least 122 970 CA Notify users as contacts through this process. Contact identification occurred a median of 4 days after symptom onset or specimen collection date of the user who received a positive test result for SARS-CoV-2.

**Practice Implications:**

Smartphone-based EN systems are promising new tools to supplement traditional contact tracing and public health interventions, particularly when efficient scaling is not feasible for other approaches. Methods to collect and interpret appropriate measures of system performance must be refined while maintaining trust and privacy.

Digital exposure notification (EN) systems use smartphones to anonymously identify and alert individuals who have been in proximity to a person who has received a positive test result for SARS-CoV-2. Such systems can automate the identification and notification of exposed contacts, supplementing traditional contact tracing programs and serving as a nonpharmaceutical intervention aimed at curbing community viral transmission. Numerous EN systems have been designed^1,2^ and deployed globally. In April 2020, Google and Apple released the Google Apple Exposure Notification (GAEN)^3^ framework for Android and iOS devices that enables an anonymous exchange of keys among smartphone users when they are in close proximity, estimated by the strength of the exchanged low-energy Bluetooth transmissions. As of May 2022, this framework has been adopted and fully operationalized by 42 countries, excluding the United States. Within the United States, 24 states, Guam, and the District of Columbia have adopted GAEN.^4^

Evidence on the performance of EN systems has been accumulating through controlled experiments^5^ and observational studies.^6,7^ Furthermore, several reports and modeling studies have demonstrated the potential public health benefits of EN systems.^8-14^ However, any potential public health impact stems from user adoption and engagement, which seem to be influenced by the design and implementation of the EN systems.^15-17^ As such, designing for real-world use and evaluating key performance indicators (KPIs)^18,19^ of the EN system is critical to assessing its impact.

California was an early adopter of Exposure Notification Express, a streamlined version of the GAEN framework. In September 2020, the state initiated a pilot of Exposure Notification Express (branded CA Notify) at the University of California San Diego and University of California San Francisco. The successful pilot prompted a statewide launch on December 10, 2020, at the peak of the winter COVID-19 surge in California.^20^ During this time, daily case counts in California exceeded the capacity of traditional contact tracing efforts, which impeded timely identification and notification of all contacts.

In this study, we present a proposed KPI framework for CA Notify along with lessons learned and future directions for the implementation of GAEN-based systems.

## Materials and Methods

### Design Process

Deploying CA Notify for people residing in California involved a human-centered design approach of iterative development and codesign with broad, diverse engagement from the community and continuous vendor feedback. We deployed and tested prototypes (touchpoints, communications language, visuals, workflow) of increasing sophistication during the pilot phase, which informed the CA Notify system configuration and final service for statewide launch. The resulting service model and current communications and designs can be found at canotify.ca.gov. Data, which contained no personally identifiable information, were shared between the University of California San Diego and California Department of Public Health by a data use agreement. The CA Notify design process did not involve research with human subjects as per 45 CFR 46.

### User Journey

**Figure 1.**
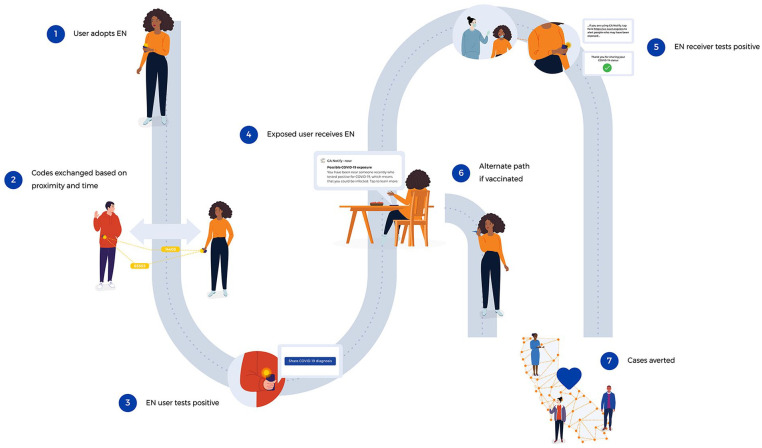
CA Notify service model and user journey. CA Notify is a digital exposure notification (EN) system in California that uses smartphones to anonymously identify and alert individuals who have been in proximity to a person who has received a positive test result for SARS-CoV-2. The system functions as follows: (1) a user activates CA Notify on one’s smartphone; (2) activated smartphones exchange keys when they are close for a defined period; (3) users who receive a positive test result for SARS-CoV-2 then receive a code that can be entered into the system; (4) inputting codes leads to notifications to users who have been in contact with a person who received a positive test result for SARS-CoV-2; (5) if a person who received an EN gets tested and has a positive test result, the user is encouraged to isolate and is sent, and can input, a code to the system, continuing the alerting cycle, or (6) a vaccinated user may choose only to monitor for symptoms and isolate if symptoms occur. The overall process leads to (7) protection of others in the community. The process depicted in the figure reflects the CA Notify functionality as well as isolation and quarantine guidance at the time of manuscript submission. The CA Notify service model and user journey are licensed under a Creative Commons Attribution–Non-commercial 4.0 International License (2021). Published with permission of CA Notify.

Users of CA Notify who receive a positive test result for SARS-CoV-2 in California can voluntarily use a unique code, which results in the anonymous alerting of other CA Notify users who came in close contact with them (Figure 1). These codes are generated automatically for >90% of all electronic laboratory records with polymerase chain reaction–positive test results in California and distributed to users via text message. When a code is sent to a CA Notify user, the user may choose to use the code by first clicking on a unique URL link that expires in 24 hours (code claimed) and then authorizing the CA Notify system to share the code with the national key server hosted by the Association of Public Health Laboratories (code used). Authorizing use of the code then leads to the release of EN alerts for other CA Notify users who have been in close proximity to the infected individual. Users who receive an EN alert are directed to a CA Notify webpage and provided public health information and directions on current guidance, such as recommendations for testing, wearing a face mask, and, if applicable, quarantining.

### Key Performance Indicators

We defined 6 KPIs to track, along with the associated data sources, metrics, methods, and limitations for calculation or estimation. Given data constraints imposed by privacy policies, several KPIs are an estimation through indirect methods (Table 1).

**Table 1.** Key performance indicator metrics, definitions, data sources, and limitations of CA Notify^a^

Key performance indicator: measurement metrics	Definition of metric	Method of calculation or estimation	Limitations	Data source
**Adoption**				
Estimated number of unique CA Notify activations	Estimated number of CA Notify smartphone activations	• The cumulative sum of estimated iOS and Android users who ever activated CA Notify. • iOS: Initial activation count was based on request to log on the iOS EN consent screen. A 0.8 multiplier (provided by the iOS team) was applied to adjust for actual activation rate. • Android: The number of downloads was derived from Google Play daily. A 0.95 multiplier (provided by the Android team) was applied to adjust for actual activation rate.	• Using activations as a proxy of adoption overestimates the true value. • People who are not residents of California might have also activated CA Notify. • A user may inactivate and reactivate or purchase a new cell phone.	• iOS: University of California San Diego Health server • Android: Google Analytics
**Retention**				
Estimated number of active daily CA Notify users	Estimated number of users who adopted and currently have CA Notify operating on their smartphones	Future estimates may use privacy-preserving analytics software developed by Google and Apple.	The actual number of active daily users cannot be calculated because of current privacy constraints. The introduction of an opt-in Electronic Notification Privacy-Preserving Analytics service may allow for indirect estimates.	Currently unavailable
**Use**				
Number of codes generated	The number of unique codes generated for users with positive COVID-19 test results	Sum of codes generated for users with positive COVID-19 test results reported in California in a given period	Codes are generated only for specimens collected and results reported in California. Codes are generated for each positive test result that is reported in California, even if the individual has recently had another positive test result and regardless of whether the individual is a CA Notify user. An out-of-state resident can participate but must have activated CA Notify and received a positive test result while in state. Such users would receive a code even if they left the state, and if they used a code, ENs would be generated for any close in-state contacts.	Electronic Notification Code Verification Server
Total number of codes claimed	The number of positive test result codes claimed by CA Notify users	Sum of codes claimed when CA Notify users click on the URL with an embedded code in a given period	—	Electronic Notification Code Verification Server
Number of codes claimed, estimated proportion	Estimated proportion of CA Notify users who received a positive test result for COVID-19 and clicked on the URL link (with an embedded code) distributed via text messages	The numerator is the number of codes claimed in a given period. The denominator is the statewide total number of positive COVID-19 test results reported during the same period multiplied by the cumulative proportion of state residents who have activated CA Notify.	The number of active daily CA Notify users cannot be calculated because of current privacy constraints. The cumulative sum of California residents who have activated CA Notify likely overrepresents the proportion of California residents with CA Notify activated during the given period.	Electronic Notification Code Verification Server
Total number of codes used	The number of unique codes shared with CA Notify to trigger EN alerts	The sum of codes used (shared) with CA Notify to trigger sending of EN alerts to other likely exposed CA Notify users during a given period.	—	Electronic Notification Code Verification Server
Number of codes used as a proportion of claimed codes	Estimated proportion of CA Notify users who shared codes after claiming codes	The numerator is the number of codes shared with CA Notify to trigger EN alerts during a given period. The denominator is the number of positive test result codes claimed by CA Notify users during a given period.	—	Electronic Notification Code Verification Server
Number of codes used as a proportion of all generated codes	Estimated proportion of CA Notify users who shared codes among all generated codes, regardless of whether the codes were claimed	The numerator is the number of codes shared with CA Notify to trigger EN alerts during a given period. The denominator is the statewide total number of positive COVID-19 test results reported during the same period multiplied by the cumulative proportion of state residents who have activated CA Notify.	—	Electronic Notification Code Verification Server and California state public data
Code use timeliness	Number of days between date of specimen collection for a positive test result or symptom onset (whichever occurs first) and code sharing to trigger EN alerts for other CA Notify users	The number of days between date of specimen collection of a positive test result or self-reported symptom onset (whichever occurs first) and date that CA Notify user shared a code	—	Electronic Notification Code Verification Server
**Identification**				
ENs acknowledged	Number of visits to the informational website by CA Notify users after receiving an EN alert	This is an indirect measure. However, the CA Notify informational website can be accessed only from a link provided to CA Notify users receiving an EN alert.	This number is an aggregate count of visits to the CA Notify URL during the study period, which does not account for an individual accessing the URL multiple times or forwarding the URL to other people who are not contacts. The count may also underreport the value, as some users may receive an EN, get tested, and follow protective behaviors without navigating to the website.	Google Analytics
**Behavior**				
Protective behaviors	Positive protective behaviors associated with CA Notify engagement, including quarantining, testing, isolating, and wearing face masks, depending on vaccine status and symptoms	Assessment of protective behaviors could not be achieved at the time of report. Ongoing efforts to include prospective and retrospective surveys have since been implemented.	These data cannot be directly measured from the current system configuration and require methods such as surveys to provide estimates.	Currently unavailable

Abbreviations: —, does not apply; EN, exposure notification.

a CA Notify is a digital EN system in California that uses smartphones to anonymously identify and alert individuals who have been in proximity to a person who has received a positive test result for SARS-CoV-2.

This study included system activity data generated by CA Notify from December 10, 2020, to July 1, 2021. We aggregated these data and reported them as KPIs, which were estimated to the extent possible, given privacy constraints. The data were then made accessible to public health authorities through a dashboard, updated daily, that included additional contextual information such as case rates and demographic information.

We modeled impact, cases, and deaths averted by using the method from Wymant et al^8^: specifically, their result of a 0.79% decrease in forecasted cases for every 1% increase in adoption of the EN system. We used the model assumption from Wymant et al of a minimum 15% of the population needing to adopt the EN system for it to have an effect, and we assumed that the secondary attack rate, level of quarantine adherence, and transmission dynamics, as well as other background interventions, policies, and protective behaviors, in California are similar to those in the United Kingdom. We estimated the number of predicted deaths averted by calculating a state death rate from the total number of COVID-19–related deaths and the total number of cases and then multiplying that value by the predicted number of averted cases. We calculated the predictions using data from December 11, 2020, to July 1, 2021 (unpublished data, California Department of Public Health).

## Results

A total of 10 910 971 smartphone activations of CA Notify (Table 1) occurred during the study period. A total of 1 654 201 codes were generated for individuals with a polymerase chain reaction–positive test result for SARS-CoV-2 and sent to their registered cell phone numbers, without knowledge of their CA Notify user status. For system use, 446 634 (27%) codes were claimed by CA Notify users, and 68 107 codes were used (15% of 446 634 codes claimed) during the study period.

During the study period, 110 497 CA Notify users were identified as contacts by measuring website visit activity. For each person who received a positive test result for SARS-CoV-2 and used the code, the system generated an average of 2 exposure alerts (contacts notified) during the study period (ENs acknowledged/codes used; Table 1).

**Figure 2.**
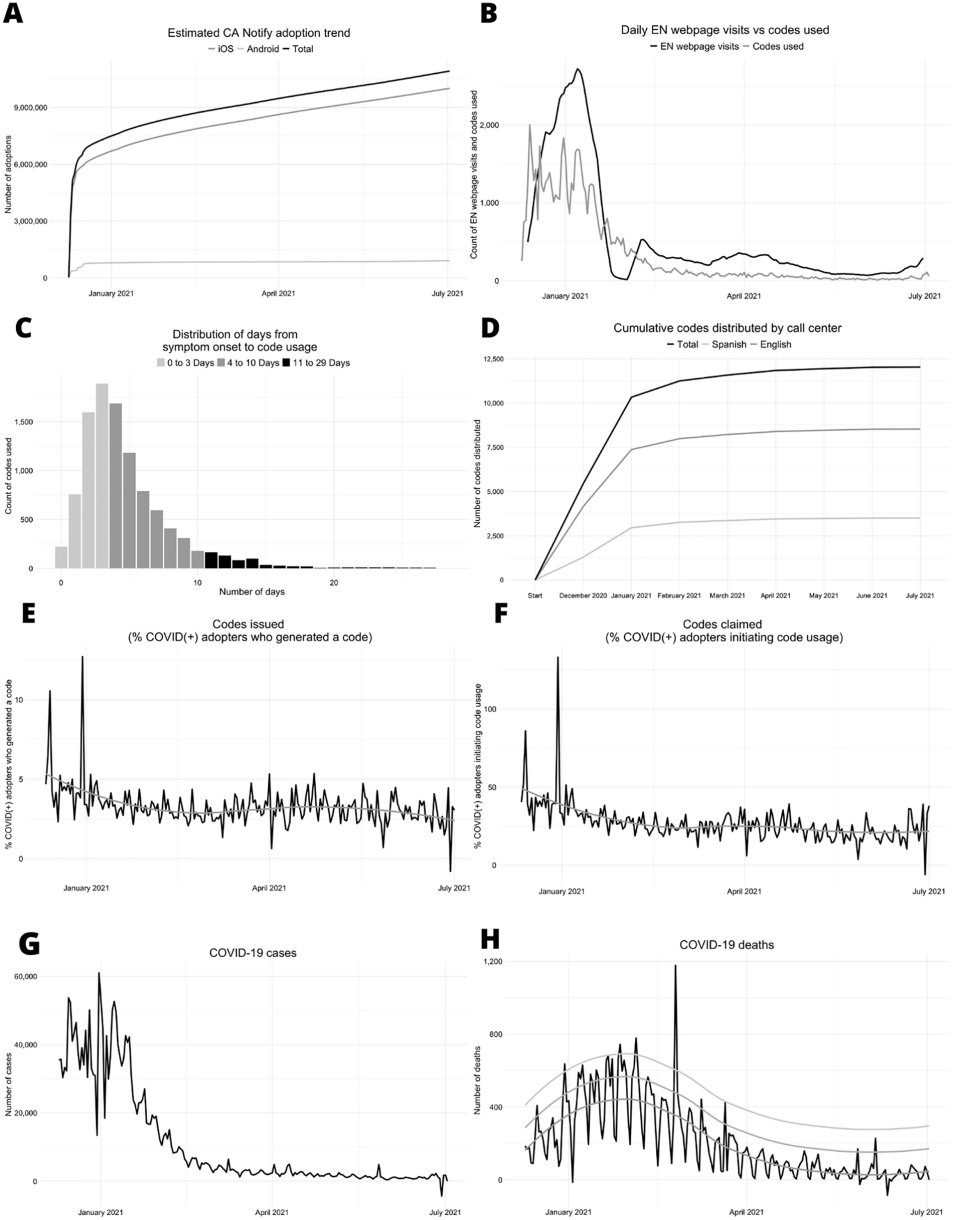
Summary of key data available for monitoring and evaluating the CA Notify system. A, Adoption (estimated by total activations). B, Number of EN page visits (note brief outage in measurement but not the system) and codes used. C, Number of days from symptom onset to code use. D, Number of cumulative codes distributed by call center by language. E, Percentage of EN users who used a code. F, Percentage of EN users initiating code use. G, Number of COVID-19 cases in California during the study period. H, Number of deaths in California during the study period. CA Notify data sources are listed in Table 2. California cases and deaths were obtained from the California Department of Public Health website.^20^ CA Notify is a digital EN system in California that uses smartphones to anonymously identify and alert individuals who have been in proximity to a person who has received a positive test result for SARS-CoV-2. Abbreviation: EN, exposure notification.

The mean (SD) number of days from symptom onset/positive test specimen collection date to code use (4.7 [3.4] days) (Figure 2) was empirically less than the mean number of days required for contact notification via traditional contact tracing methods used by California’s contact tracing program (7 days).

## Discussion

While developing, deploying, and monitoring the CA Notify system, we operationalized our lessons learned into a continuous improvement framework, which we applied to our KPIs. We present public health implications and actions to mitigate challenges specific to our KPIs (Table 2). We identified data requirements for what type of data must be gathered, during what period, by what means, and with what frequency.

**Table 2.** CA Notify key performance indicators: observations and implications, December 12, 2020–July 1, 2021^a^

Dimension	Observations	Calculations and estimates	Implications
Adoption (smartphone activations)	• Access restricted to smartphone users who have compatible operating systems (iOS or Android) and are able to overcome potential barriers of language, readability, and usability. • Usability and trust scales across focus group populations suggest that acceptability varied. • Activations peaked in the first week after release.	• Potential CA Notify access is about 85% of population (upper limit estimate of eligible smartphone users based on Pew study).^21^ • Actual activations: about 29% of state residents (10.9 million) estimated to have activated CA Notify based on 9 998 611 iOS and 912 360 Android counts. • Acceptability: High trust scores from focus group testing.	• Adoption highest in the launch phase when Apple pushed availability alerts. • Ongoing engagement strategies are needed. • Key groups remain disenfranchised or have access issues.
Retention (active users)	User choices: (1) maintain CA Notify, (2) turn off CA Notify, (3) turn on/off.	Under investigation.	• Engagement strategies required to improve adoption and retention especially among groups at high risk for disease or serious outcomes. • Need for methods to accurately measure active daily users and key risk factors.
Use (appropriate actions followed)	Code use: users must claim and use (share) codes to release EN alerts.	• Number of codes claimed: 446 634 (27% of codes issued) • Number of codes used: 68 107 (15% of codes claimed)	• Codes claimed map to adoption numbers for California population. • Code use can be influenced by improved SMS and more frequent touchpoints.
Identification (identification of new contacts/cases)	Estimated by number of codes used and number of EN page visits.	• Number of EN page visits: 122 970 • Mean code use interval: 4.7 d • Mean (95% CI) [SD] identification ratio: 1.8 (0.2-11.3) [1.76]	• Estimated identification rate is higher than using traditional contact tracing in California. • Likely discovers contacts that do not overlap with traditional methods.
Behavior (change in behavior after receiving a notification)	An EN is received and read, leading to recommended/expected behavior change/action.	Direct assessment of behavior change is limited because of privacy constraints.	• Privacy-preserving architecture can limit measurement of key performance indicators. • Balances must be struck between these dimensions. • Prospective and retrospective surveys can be deployed to gather more information in an opt-in manner.
Impact (measuring public health outcomes and implications)	• Integration of key performance indicators. • Model estimates of cases reached, cases averted, time to recognition change, hospitalizations, deaths, and cost.	Appropriate models that accurately predict/determine impact are lacking for US implementation of EN.	• Preliminary models indicate likely impact; however, none is tailored to the US experience with EN. • Further models are needed.

Abbreviations: EN, exposure notification; SMS, short message service.

a CA Notify is a digital EN system in California that uses smartphones to anonymously identify and alert individuals who have been in proximity to a person who has received a positive test result for SARS-CoV-2.

Use of the Exposure Notification Express version of GAEN led to challenges in estimating KPIs. These challenges are not unique to CA Notify and stem from the intrinsic privacy-preserving nature of the platform. EN systems in countries such as the United Kingdom enable the direct calculation of critical metrics, such as the secondary attack rate. However, in California, these approaches remain to be implemented, and future consideration will need to balance privacy and public acceptability with the need for system monitoring, security, and impact measurement, thereby establishing benefits over harms to inform policy decisions.

Despite innate limitations introduced by a privacy-preserving policy of minimal data collection and by intrinsic privacy measures, we attempted to provide a structured KPI framework and the context needed to interpret the value and limitations of the data. For example, using activations as a surrogate for adoption presents an upper bound only and is itself potentially biased by out-of-state adopters, those who uninstall and reactivate the app, or those who upgrade to a new cell phone. Related to this limitation, estimation of retention, or the number of active daily users, should be achievable with opt-in privacy-preserving analytics; however, the system developed for Exposure Notification Express was not immediately available at the time of launch and infuses privacy-preserving noise (random numbers) into the system to further anonymize data. To date, this injection of noise has made estimating active daily users difficult and detection of SARS-CoV-2 among EN users not feasible.

Measuring the influence of CA Notify on public health outcomes (cases, hospitalizations, and deaths) through the estimated KPIs and mathematical models is a topic of ongoing research. The final KPI, an estimate of impact, should be considered more preliminary than the first 5 KPIs and is the subject of ongoing investigation. The uncertainty, in addition to that which is inherent to the mathematical models, is due to a lack of accurate estimates for key model input metrics, including the true number of active daily users, the percentage of those alerted who are infected with SARS-CoV-2, and whether they follow the protective behaviors (eg, testing, self-quarantine) required for impact. In the absence of such metrics and models for California to date, if we (1) apply the model developed by Wymant et al^8^ to CA Notify adoption data and (2) make a series of assumptions that secondary attack rate, quarantine adherence, and transmission dynamics, in addition to other background interventions, policies, and protective behaviors, in California are similar to those in the United Kingdom, then we would have averted >31 000 cases and >600 deaths in the state from December 11, 2020, to July 1, 2021. While estimates are speculative, they are encouraging evidence to support extending and expanding our evaluation efforts. The applicability of external models to other programs (eg, European models for US EN programs) remains controversial because of differences in system design, implementation characteristics, and data availability. Therefore, we are developing an agent-based model and aggregating survey and contact tracing data to estimate the impact of CA Notify on cases and deaths.

## Practice Implications

In practice, we must be careful not to overinterpret our estimated KPIs considering assumptions required due to limited data, as previously discussed. Our study had important limitations pertaining to data access and reporting for CA Notify. While activations reached substantial levels (up to 40% of eligible users), these estimates likely reflect overcounting. Higher EN adoption and mitigation of factors leading to barriers to access and disparities in availability and use must be pursued. These concerns are the topic of ongoing investigation. Similarly, more granular estimates of active daily users are lacking, and for these users, it is difficult to directly ascertain the type of behavior changes (eg, testing, wearing face masks) taken by individuals who receive a notification alert. The impact of CA Notify is predicated on such changes in behavior, and we are currently seeking to better assess these changes by using voluntary prospective and retrospective surveys. Finally, our estimate of impact was limited to the use of a published model that has not been validated outside its intended context and, thus, should be considered preliminary. Future models that are bespoke to EN system implementation in California are in development to address these shortcomings.

Several initiatives are being undertaken to address data availability due to the privacy-preserving features of CA Notify and our estimation limitations, including conducting multiple surveys of California residents, opt-in prospective repeated measures surveying CA Notify users, and targeted retrospective analyses. Furthermore, to the best of our ability, adoption and use barriers have been minimized by applying a participatory design approach through each phase of our operations. Notably, this human-centered design approach brings together developers, users, public health, and other stakeholders in the community to negotiate complex issues of communication, accessibility, value proposition, and policies for implementation. While we will continue to refine our approach, we encourage others to consider these methods in their COVID-19 public health programs. In our case, engagement of diverse groups, in multiple languages, drawn from state government and university communities and through established community-based organizations provided important insights to reduce barriers and foster adoption. Still, formative work did not include a sufficiently representative sample of residents of California; as such, it will be important to observe trends in adoption as CA Notify is promoted across the state. Moreover, we are uncertain what motivates an individual to claim and then use a code (behavior), which will notify others who may have been exposed. Measures to protect privacy and anonymity were prioritized, which limited data utility. To determine whether prioritizing privacy is a public preference (by using Bluetooth rather than global positioning system location coordinates and avoiding collecting data identifiers), we will systematically survey residents of California to gather behavioral data and privacy preferences.

During the study period, up to 30% of California’s population (10.9 million activations; state population, 39.4 million) and about 40% (assuming 33 million) of all smartphone users^21^ activated the CA Notify system, with EN alerts typically occurring faster than traditional contact tracing and notification efforts and reaching a greater number of contacts per index case. We believe that this level of adoption was facilitated by ease of activation (particularly for iOS instances) and availability alerts and, in part, because of the process of engagement and stakeholder input into the system design. The disproportionately higher adoption by iOS users as compared with Android users is notable and has been observed in other settings.^8^ These data provide some evidence that a system-integrated solution, rather than a stand-alone app, may more effectively promote technology adoption.

Finally, there was a close and collaborative feedback loop throughout planning and deployment of CA Notify among academic implementing partners, public health officials, Google, Apple, and others. This partnership allowed design ideas and communications to flow from formative research by University of California investigators to industry partners, while focus groups tested new functionality and public health officials provided requirements and direction in real time. This close collaboration was a key to project success and produced lessons learned that can benefit others who are developing digital EN programs.

Our experience suggests that digital EN systems offer a synergistic addition to nonpharmaceutical interventions available to public health authorities, particularly extending the reach of traditional contact tracing methods during surges of infection. A growing corpus of literature,^9-11^ notably focused outside the United States, and our preliminary investigations of impact show the potential for such programs. As others have noted, global smartphone adoption rates are high, even in the most remote and resource-constrained settings (where novel pathogens often emerge), providing a potential global sensing and action network capable of functioning within and beyond the scope of public health authorities. Future work and coalitions are needed to create readily deployable, transparent, community-supported instances, as well as standardized methods to govern, evaluate, and evolve the system, considering the costs, benefits, and ethical design with privacy-preserving protocols. EN is a compelling tool during surges of COVID-19 and SARS-CoV-2 variants and may help to prevent or mitigate future pandemics.
